# Bullying Experience among Adolescents with a Turkish Migration Background in Germany: Ethnic Class Composition, Integration, and Religiosity as Protective Factors?

**DOI:** 10.3390/ijerph17134776

**Published:** 2020-07-02

**Authors:** Sarah Demmrich, Semra Akgül

**Affiliations:** 1 Cluster of Excellence ‘Religion & Politics’, Chair of Sociology of Religion, University of Muenster, 48143 Münster, Germany; 2 Centre of Clinical Psychology and Rehabilitation (ZKPR), University of Bremen, 28359 Bremen, Germany; akguel@uni-bremen.de

**Keywords:** bullying, migration, Turkey, ethnic class composition, acculturation, religiosity, depressiveness

## Abstract

Bullying is a worldwide problem that has serious effects on the mental health of both victims and perpetrators. Although bullying seems related to increasing globalization and migration, it has seldom been researched in this context. This exploratory study examined bullying experiences and related depressive symptoms among a sample of adolescents with a Turkish migration background in a German school context (*N* = 103, 56.7% female, *M_Age_* = 16.17, *SD_Age_* = 1.36). The study focuses on three migration-related variables as potentially salutogenic factors: Ethnic class composition, acculturation, and religiosity. While the ethnic class composition did not show any effect, an integration acculturation strategy and religiosity proved to be protective factors against bullying experience. The negative prediction of integration on depressive symptoms was not consistent throughout the multivariate analyses. The results are discussed against the background of new impulses for bullying intervention programs for this vulnerable group of adolescents from a Turkish migration background.

## 1. Introduction

Bullying is a prevalent issue [1,2] accompanied by related mental health problems for both victims and perpetrators, including symptoms of depression, anxiety, and even suicidality [3]. Bullying can be defined as a negative, deliberate behavior that is repeated over time, is performed by one or more individuals, and is directed against a person with the aim of harming him/her; thus, it involves a power imbalance between victim and perpetrator [4]. This negative behavior encompasses threats, mockery, verbal abuse, and beating (pushing, kicking, punching), as well as cyber bullying, a newly developed form of aggressive behavior performed using modern information and communication technologies [5,6]. Generally, an individual can experience bullying by actively bullying (perpetrator), being bullied (victim), or both (victim–perpetrator) [7] (For clarity throughout the study, we use the term bullying experience to refer to all three described phenomena while the terms bully and bullying are used when describing the perpetrator’s behaviors.)

Children and adolescents with bullying experience—whether perpetrators, victims, or victim–perpetrators—report negative long-term mental health consequences that extend into adulthood, including interpersonal problems [8] and depression [9]. Specifically, they exhibit low self-esteem, increased loneliness, anxiety, a higher rate of substance abuse, and delinquent behavior, as well as suicidal intentions [1,6,10,11]. The summary of existing studies [12] suggests that victim–perpetrators show the highest rates of psychological impairment, such as internalized and externalized problems, lower performance at school, interpersonal problems, and greater suffering from the victimization experience. In addition, some sociodemographic variables were identified as significant risk factors, especially gender (boys are more likely to bully, whereas girls are more likely to become victimized) [13] and low socioeconomic status (SES; increases risk of becoming a perpetrator, a victim, or a victim–perpetrator [14,15].

However, not all children and adolescents develop adaptation problems after having bullying experiences [16]. Those who, on average, have more resources that protect them from negative short- and long-term bullying consequences can go on to develop normally. Previous studies have underscored a high intelligence quotient, good academic achievement, high self-esteem, and self-efficacy as important protective factors [8,16,17,18]. In addition to these internal components of resilience, external components like a stable family, positive peer influence, and a strong bond to school are seen as protective factors for bullying [19]. Connectedness to the school itself, as well as between pupils and teachers, also increases the effectiveness of bullying prevention programs [20].

Going beyond these general salutogenic factors, this study aims to identify specific protective factors for a generally vulnerable group: Adolescents with a Turkish migration background in Germany. By examining three potentially protective factors for this group, i.e., ethnic class composition, acculturation strategies, and religiosity, this study fills a gap in the research on bullying experience in the context of migration.

### 1.1. Bullying in the Context of Migration

Some scholars attribute the prevalence of bullying to globalization and migration, and the inevitably greater presence of children and adolescents with a migration background in schools [21,22,23]. However, research on bullying in the context of migration is still in its early stages [24]. Germany plays a very important role in this context, having 17 million residents with a migration background. The largest sub-category in this group is formed by the approximately three million people that have a Turkish migration background [25]. The history of mass migration from Turkey began with the German-Turkish Recruitment Agreement in 1961 [26] and today’s adolescents with a Turkish migration background constitute the second, third, and even sometimes fourth generation. In contrast to the first-generation migrants from Turkey, these subsequent generations feel strongly attached to, and at home in, Germany [27].

From the perspective of developmental psychology, adolescents with a migration background not only face the usual adolescent developmental tasks but also additional migration-specific tasks. These additional tasks include acquiring a new language, translating language and culture for older family members [28], and internalizing the norms and values of the majority culture due to the high integration pressure of the school environment [28,29]. Moreover, an acculturation-specific dimension is added to the regular developmental tasks, which emphasizes the contrast between an individualistic culture (Germany) and a mainly collectivistic culture of origin (Turkey) [30]. This means, in this context, the regular developmental task of identity formation [31] becomes a question of cultural identity; the typical adolescent task of forming a system of norms and values [32] is more complex by virtue of being linked with (Muslim) religiosity and collectivism. Furthermore, the individualistic-connoted adolescent developmental task of the achievement of emotional independence from parents and other adults [33] is usually postponed into adulthood among youths from collectivistic families [29].

The school context is an excellent environment for studying the acculturation of children and adolescents as it is a place where children and adolescents with different cultural–religious backgrounds regularly come together and have the opportunity to socialize. According to previous studies, children from ethnic minorities experience less bullying at schools which are characterized by a high proportion of foreign pupils [34,35]. Consistent with this finding, it has been repeatedly demonstrated that when ethnic class composition at school is quite homogenous, the members of minority ethnic groups experience more victimization and the children of mainstream society are more aggressive [34,36,37]. In contrast, a study of 11 countries, including Germany, by [23] returned the opposite results, suggesting that both migrant and non-migrant adolescents bully more when there is a large number of adolescents with a migration background at a school, however the effect was rather small. Additionally, [38] could neither find a significant influence of school’s ethnic composition on victimization nor a moderation of it on the relationship between victimization and well-being. In sum, the ethnic composition of a class or school as a predictor of bullying and victimization experience remains controversial. Furthermore, in Germany, the level of contact between pupils within a school class is usually much greater than it is between different classes in the North America school system, on which many of the above-mentioned studies are based. Hence, our focus is on ethnic class composition rather than school composition.

In addition to the ethnic class or school composition and the previously elaborated acculturation-specific connotation of developmental tasks, acculturation strategies might also play a crucial role in the process of adolescents’ bullying in the context of migration. Acculturation is defined as the way individuals balance the importance of maintaining their culture of origin and the importance of the contact with members of and identification with the majority culture [39]. This balance can result in four outcomes: Integration means trying to adapt to the majority culture while maintaining elements of the culture of origin at the same time. The reserved pattern, in which both the host and the original culture are rejected, is called marginalization, and is seen as a pathogen factor in the acculturation process as it is often accompanied by depressive symptoms and an instable identity [40]. When the individual importance of the maintenance of the culture of origin is high and the importance of the culture of the host society is low, [39] speaks of separation. The reversed pattern, in which the culture of origin has no importance in an individual’s life but the importance to become a part of the majority culture is high, is called assimilation.

Previous studies have shown that the acculturation strategy of integration acts as a salutogenic factor and is linked to low rates of depression and a high level of success at school [41], as well as less aggressive behavior concurrent with a higher level of prosocial behavior [42]. That these results regarding integration as a protective factor can be transferred to bullying was demonstrated in a study of a multi-ethnic sample of Australian adolescents [3]. Against the backdrop of these studies, adolescents with a Turkish migration background in Germany might be vulnerable [6,24] since the most often applied acculturation strategy among Turkish migrants, and their descendants in particular, is separation [43,44]. Additionally, Turkish migrants and their descendants are marked by a lower than average socioeconomic status (SES) [45], which can contribute to their vulnerability to bullying experiences and their negative mental health consequences [15].

### 1.2. Depressive Symptoms, Bullying, and the Role of Religiosity

In addition to the negative mental health consequences of bullying, victimization, and bullying–victimization previously mentioned, depressive symptoms are the specific focus of this study [9] as depression is the most common mental disorder experienced during adolescence [46], with girls scoring higher on depression scales than boys [47]. With specific regard to depressive symptoms, victims, but also the victim–perpetrators, show higher depressive symptoms than both perpetrators and individuals without bullying experience [6].

With specific regard to adolescents with a Turkish migration background in Germany, dealing with the additional migration-specific tasks on top of regular adolescent developmental tasks [48] can cause acculturation stress, which is probably a risk factor for depressive symptoms [49]. However, there also seem to be additional protective factors against depressive symptoms among adolescents with a migration background that go beyond the relationship between the ethnic composition of their class/school and bullying experience. One of these might be religiosity, which has not received, to the best of our knowledge, sufficient attention in the context of previous studies investigating bullying experience at school. Generally, religiosity remains an important part in the lives of Turkish adolescents [50,51] and this remains true for adolescents with a Turkish migration background [52,53].

In general, religiosity, in regard to individual lived religion (which encompasses religious behavior, cognition, and emotion), has been proven to be a predominantly salutogenic factor among Muslims [54,55,56] showing positive links with, for example, life satisfaction and happiness [50,57]. More specifically, the high religiosity of Turks was found to be a protective factor against depressive symptoms [58] and suicidality [59]; furthermore, it was shown to play a positive role in prosocial behavior [60]. The relation between religiosity and aggressive/violent behavior among adolescents seems to influence bullying experience but is empirically controversial: While a negative link could be empirically shown in Muslim-majority countries [61], no correlation between Muslim religiosity and violent behavior, not even a positive one, was revealed in the context of migration to Germany when confounding variables were controlled [62]. Whether these results on the links between religiosity and depressive symptoms or aggressive/violent behavior can be generalized to religiosity and bullying experience is a central question of this study.

An extensive literature search uncovered only four studies dealing with religiosity and bullying experience. Using religiosity as an outcome variable, [63] found a link between retrospectively reported childhood bullying experience and adult religious/spiritual well-being in a cross-sectional study performed among a sample of hospitalized adults in the USA. By emphasizing the missing link between religiosity and bullying experience, [64] found support for a moderating effect of religiosity on the relationship between substance use and different forms of victimization among a sample of Lebanese adolescents in their very recent study. Additionally, there are two studies that analyzed variables related to religiosity as predictors of bullying. Firstly, according to [65], spiritual well-being served as a protective factor against bullying and victimization among Slovakian adolescents. Secondly, in their longitudinal study in England, [66] used religious affiliation to show that adolescents who indicate no religious affiliation score significantly higher on victimization experience at school than adolescents who belong to a religion. However, none of the studies focused on religiosity as a protective factor against bullying experience in the context of depressive symptoms.

### 1.3. The Current Study

Our study aims to research the influence of gender, self-reported SES, ethnic class composition, acculturation strategies, and religiosity on bullying experience, as well as the possible link to depressive symptoms among a sample of Turkish adolescents living in Germany.

Previous studies suggest that ethnic class composition is a crucial factor when researching bullying and victimization [36], however these results are controversial [23]. Based on the findings of the majority of these studies, the first aim of the research presented herein is to test the hypothesis that adolescents with a Turkish migration background are more likely to experience victimization when they are the minority in the school class and bully more when they make up the ethnic majority of the school class.

Regarding acculturation strategies, we want to further test whether the finding that the integration acculturation strategy of the multi-ethnic sample in Australia was negatively linked with bullying and victimization experiences [3] can be generalized to Turkish adolescents living in Germany.

In general, religiosity shows positive associations to prosocial behavior [60] and negative relationships to depressive symptoms [58], but contradicting links with aggressive/violent behavior [61,62]. However, the specific role of religiosity in bullying experience remains a research desideratum addressed by our study. Finally, we raise the question which of the variables under consideration (ethnic class composition, acculturation strategies, religiosity) contribute most to the prediction of bullying, victimization, and bullying–victimization when controlling for age, gender, and SES.

Depressive symptoms are highly correlated with bullying experience [9] and migrant adolescents seem to be more vulnerable to developing depressive symptoms due to acculturation stress [49]. However, ethnic class composition, integration as an acculturation strategy, and religiosity seem to serve as protective factors regarding the link between bullying experience and depressive symptoms. Therefore, our last research aim is to investigate whether these three variables demonstrate a buffering effect on the relationship between bullying experience and depressive symptoms in our sample.

## 2. Method

### 2.1. Procedure

The study was conducted in line with the ethical regulations of the psychology department of University of Bremen, and data were collected between June and August 2019. Participants were recruited from special interest groups and social clubs with strong associations to Turkish migrants and their descendants, such as the youth associations of Turkey-based mosques (DITIB, *Milli Görüş*; for a deeper insight into these organization see [67]) as well as secular Turkish-culture associations in five different cities in Germany (Berlin, Bremen, Hamburg, Rothenburg, Neumunster). The second author of this study, who is fluent in Turkish as well as in German, administered the paper-pencil questionnaires in the German language to these non-clinical participants in group settings on the premises of the respective associations. In order to ensure space and the privacy of answers, the groups consisted of approximately five participants who each filled out the questionnaire at the same time. The second author was also available for questions and explanations, but no major issues around understanding occurred. Inclusion criteria required participants to have a Turkish migration background and be a current school student. That these criteria were met was ensured by the first questions in the questionnaire (see measurement section; no participants were excluded since all had met the criteria). All parents gave their consent and participation in the study was voluntary. Anonymity was ensured.

### 2.2. Measurements

Sociodemographic and socio-religious variables: Besides age and gender, the self-reported financial situation of the family was used as an indicator for SES: “According to your opinion, how is your family’s financial situation?”, the answer scale ranged from 0 = very bad to 4 = very good. The migration history of the family was collected (Who migrated first to Germany? father/mother = second generation, grandfather/grandmother = third generation; great-grandfather/great-grandmother = fourth generation), as was the type of school the participant attends (coded according to International Standard Classification of Education ISCED-2011: 2 = lower secondary, 3 = upper secondary including vocational training). Finally, the participant was asked for their religious affiliation using a single-choice answer format (Sunni, Alevi, Christian, others, none).

Bullying and victimization: The Bullying Others and Victimization Scale [68] defines bullying in the scale instructions as unfriendly behavior towards schoolmates who are not able to defend themselves, including kicking, pushing, threatening, harassing, teasing, and excluding. This reliable and valid scale was used in our study not only for its applicability for adolescents but also for immigrants from diverse cultural backgrounds. This measurement including its instruction was translated internally by the authors of this study taking into consideration the recommendations of a German version by [69] and using independent back and forth translations until a satisfactory agreement was achieved [70]. The scale asks for the frequency of general, verbal, social, and physical bullying (e.g., “This school year: How often have you bullied/hassled other pupils at school by teasing them?”) as well as victimization (e.g., “This school year: How often have you been bullied/hassled at school by being teased?”) by using four items for each subscale. In order to meet the current state of research [5], we added a fifth item for each subscale, which adds the aspect of cyber bullying (“This school year: How often have you bullied/hassled other pupils at school by mean texts, mails, videos, or photos?/This school year: How often have you been bullied/hassled at school by mean texts, mails, videos, or photos?”). Answers were given on a frequency scale ranging from *0 = never* to *3 = daily*. Cronbach’s alphas amounted to *α* = 0.83 for the bullying and victimization subscale, respectively (original: Between *α =* 0.72 and *α =* 0.91). Scoring high on both subscales indicates being a bully and a victim at the same time (victim–perpetrator); therefore, the whole scale was also taken into the analyses (*α* = 0.79).

*Depressive symptoms*: A short version of Beck’s Depression Inventory [71] was applied as a highly reliable (original: *α* = 0.90; here: *α* = 0.91) and valid measurement in German, which was also applicable to adolescents. Usually containing 20 items answered on a five-point frequency scale from *0 = never* to *5 = almost always*, one item was excluded from our questionnaire (“I don’t care about sex”) as we determined it was an invalid indicator for depressive symptoms for adolescents starting from 14 years old.

Ethnic class composition: The question “How many pupils from your class are from a Turkish background?” was answered on a five-point scale from 0 = almost all of them to 4 = very few are.

*Acculturation strategies*: The East Asian Acculturation Measure (EAAM) [72] is one of the few scales which measures all four proposed acculturation styles [39], is frequently used in student samples [73], and was in earlier studies applied to different cultures of origin and majority cultures [74]. The EAAM was internally translated as described above and, in the process, adapted for German mainstream society and minority from a Turkish migration background within it. The whole scale consists of 29 items, which measure four acculturation strategies: Assimilation (eight items, e.g., “When I am in my apartment/house, I typically speak German”; original: *α* = 0.77; here: *α* = 0.79), integration (five items, e.g., “I have both German and Turkish friends”; original: *α* = 0.76; here: *α* = 0.61), separation (seven items, e.g., “My closest friends are Turkish”; original: *α* = 0.74; here: *α* = 0.77), and marginalization (nine items, e.g., “Generally, I find it difficult to socialize with anybody, Turks or Germans”, original: *α* = 0.85; here: *α* = 0.86). Answers were given on a seven-point scale from *1 = I completely disagree* to *7 = I completely agree*. (Cronbach’s alpha for integration is on the lower limit of an acceptable reliability. After an in-depth analysis of internal consistency, the exclusion of items would not increase the alpha significantly. Instead, the main issue for this low internal consistency is most probably that this subscale tries to cover two moderately-correlated aspects of acculturation, i.e., social (making friends, feeling accepted) and cognitive (thinking, writing), with only five items in total. Other studies [73,74] reported similar reliability problems of the integration subscale of EAAM adaptations with *α* = 0.64 and *α* = 0.62, respectively.)

Religiosity: The indigenous measure of The Religious Belief Scale for Adolescents (Ergenler İçin Dini İnanç Ölçeği) was developed by [75] for the use of measuring religiosity of Turkish adolescents and translated as described above. This unidimensional scale consists of 18 items (e.g., “I believe that God hears my prayers”). Answers were given on a five-point scale, ranging from 1 = I completely disagree to 5 = I completely agree. Cronbach’s alpha is high, with α = 0.95 (original: α = 0.94).

### 2.3. Sample

This convenience sample consisted of a total of *N* = 103 adolescents with a Turkish migration background living in Germany (56.7% female). The age of the participants rangesd from 14 to 18 years (*M* = 16.17, *SD* = 1.36). While *n* = 86 were Sunnis, *n* = 7 Alevis, *n* = 6 indicated ‘other, Islam’, two additional participants indicated not feeling affiliated to any religion, and one participant identified as belonging to another religion (not further specified; *n* = 1 missing).

The majority of the sample consisted of the third generation (i.e., grandfather/grandmother migrated to Germany, 81.4%) and only 14.7% and 3.9% of participants belonged to the second and fourth generation, respectively. The sample was biased towards a high education as 76.5% indicated an upper secondary education (residual: Lower secondary education). Moreover, most adolescents rated the financial situation of their families as moderate to good (on a scale from 0 to 4: *M* = 2.73, *SD* = 0.72).

### 2.4. Data Analyses

All statistical analyses were carried out using SPSS version 26 (IBM, New York, NY, USA). For a general overview, we first performed Pearson-correlation analyses, and the specific focus on the correlations between bullying experience and three potential protective factors (ethnic class composition, acculturation strategies, and religiosity) addressed the first three research aims. In order to control for various confounding variables, three separate multiple regression analyses on bullying, victimization, and bullying–victimization were performed, which accounted for the potential protective factors as well as numerous control variables as predictors. In order to test whether the potentially protective factors predict depressive symptoms and show a buffering effect on the proposed relationship between bullying experience and depressive symptoms, a hierarchical regression analysis on depressive symptoms was performed. It included five models which successively added variables of interest to the proposed relationship while controlling for confounding variables.

## 3. Results

### 3.1. Preliminary Analysis

Providing a general overview, Table 1 displays all included variables and their Pearson correlations with means and standard deviations of the distribution of each variable in the diagonal. Regarding the sociodemographic variables, it is noteworthy that gender is related to bullying experience (but not depressive symptoms) as girls scored significantly lower on active bullying and higher on victimization experience. Moreover, education level shows a moderate negative correlation with bullying (but not victimization or the mixed type). Finally, age is confounded with assimilation.

### 3.2. Correlations between Ethnic Class Composition, Acculturation Strategies, Religiosity, and Bullying

Regarding the first three study aims, bivariate relations between the three potentially protective variables of interest and bullying experience were conducted (Table 1). While the ethnic class composition showed no relationship to bullying, victimization, or bullying–victimization, integration did show a moderate negative correlation to all of these experiences. Interestingly, marginalization, another acculturation strategy, showed the same relationships but with inverse valence. Finally, religiosity was highly negatively related to victimization and bullying–victimization, but not to bullying. However, Table 1 also indicates intercorrelations between explaining variables. The elimination of these confounding effects is addressed in the following analyses.

### 3.3. Regression Analyses on Bullying, Victimization, and Bullying–Victimization

In order to address the fourth study aim, which is to test all three potentially protective factors (ethnic class composition, acculturation strategies, religiosity) as predictors of bullying experience while controlling for sociodemographic variables, three multiple regression analyses were performed on bullying, victimization, and bullying–victimization, respectively (Table 2). First, bullying was significantly and negatively predicted by education level and integration on a moderate level, but not by ethnic class composition and religiosity, which is in line with the bivariate analyses. Second, female gender was a strong-positive predictor of victimization, but religiosity was an even stronger but negative predictor. The moderate and negative predictive effect of religiosity remained when bullying–victimization was the criterion and integration had the same effect on bullying–victimization independently. Again, the variable ethnic class composition did not serve as a predictor in the last two analyses. In total, all three regression models were significant and each explained a moderate amount of variance.

### 3.4. Hierarchical Regression Analysis on Depressive Symptoms

The last study aim addressed the question of whether ethnic class composition, acculturation strategies, and religiosity can lower the relationship between bullying experience and depressive symptoms while controlling for sociodemographic variables. For this reason, a hierarchical regression analysis was performed. Table 3 (models 2–5) shows a significant positive, medium to strong correlation between bullying–victimization and depressive symptoms that remains robust under inclusion of sociodemographic variables (although SES is continuously a negative predictor too) and all other potentially protective factors (models 3–5). This means that neither ethnic class composition (model 3), acculturation strategies (model 4), nor religiosity (full model 5) could lower this relationship. Moreover, neither the inclusion of ethnic class composition (model 3) nor religiosity (model 5) significantly increased the level of explained variance. However, including acculturation strategies in model 4 did increase the explained variance a significant amount. Independent of bullying–victimization, integration, and marginalization both played an important role in the prediction of depressive symptoms. While integration is a negative predictor in model 4, it only falls slightly under the critical value of *p* ≤ 0.05 in the full model. Marginalization appears in the last two models as a strong positive predictor of depressive symptoms.

## 4. Discussion

This study was, to the best of our knowledge, the first one that tested bullying experience and accompanied depressive symptoms among individuals who are often considered a vulnerable group on the basis of their usually lower-than-average SES [45] and additional developmental tasks related to acculturation [28,29,49]: Adolescents with a Turkish migration background in Germany. Due to the limited sample size, the resulting low statistical power, the convenience sample’s bias towards a high education level, and probably higher SES than would be found among larger samples of individuals with a Turkish migration background [45], this study must be considered exploratory. (It is also possible that the self-reported financial situation of the family underlies biases by the participant towards a higher status than the SES, in an economic sense, would otherwise indicate. This was outlined in a representative study among participants starting from 16 years old with a Turkish migration background in Germany [76].) However, gender was balanced and the fundamental results of previous studies regarding the role of sociodemographic variables in bullying experience were confirmed here. For example, girls were found to be more likely to become victims and less likely to become perpetrators once again [13], and education level was shown to relate negatively to bullying [12]. Moreover, all bullying experiences were positively related to depressive symptoms [1,6,9,10,11], with bullying–victimization as the most pronounced correlate and predictor of such symptoms [12]. Despite the small, biased sample, these replicated results underline the validity of the other findings regarding the three potentially protective factors under consideration, which showed significant results even when holding multiple other variables constant.

Considering the first factor—ethnic class composition—none of the analyses revealed significant relations to bullying experience or depressive symptoms. This does not align with our previously formulated hypothesis based on several studies that showed a protective effect of a high number of pupils with a migration background in school/class [34,36,37]. It is also inconsistent with the reverse finding by [23]. It seems that neither a high nor a low number of classmates with a Turkish migration background influences whether adolescents with such a background go through bullying experiences; therefore, this result aligns with the findings by [38]. Due to the robustness of this non-significant finding throughout all the analyses, we conclude that ethnic class composition seems not to serve as a protective factor among our sample of adolescents with a Turkish migration background in Germany. Another related variable could shed more light on these contradictory findings: Whether bullying is more harmful for an adolescent when it is performed by a same-ethnicity or other-ethnicity peer should be addressed more deeply in future studies [38].

Regarding the second factor—acculturation strategies—our sample scored high on separation, which is in line with previous studies [43], but it scored even higher on integration, which might be specific to the youngest generations of descendants of migrants of Turkish origin, who were found to be strongly attached to Germany [27]. The average score on assimilation was high, too, but declined with increasing age. As expected, adolescents from a Turkish migration background seemed to apply marginalization less often, which is a maladaptive acculturation strategy [40] and, in this study, strongly related to depressive symptoms. Regarding the acculturation strategy of highest interest—integration—adolescents with a Turkish migration background seemed to go through bullying and bullying–victimization experiences less often when they predominantly applied integration as an acculturation strategy, as was shown in a previous study in a different cultural context [3]. Although integration is not a buffering factor in the relation between bullying experience and depressive symptoms, it seems to be a protective factor against depressive symptoms. As the significance of this effect is not robust throughout the multivariate analyses, future studies with a larger sample, and therefore higher statistical power, need to replicate this finding to prove its reliability and validity. Additionally, the integration subscale’s internal consistency needs to be improved, as the low Cronbach’s alpha was not limited to our study [73,74]. Therefore, we conclude that integration seems to protect against bullying experience and also, independently, against depressive symptoms. Conversely, marginalization appears to be a risk factor for depressive symptoms but independent from the bullying experience–depressiveness link.

Finally, the last potentially protective factor—namely religiosity—showed a negative bivariate relation to victimization and bullying–victimization, but not to bullying itself, which was proved robust in the multivariate analyses. Firstly, it confirmed the bivariate results of [62], who found no relation between religiosity and aggressive behavior among a representative sample of Muslim adolescents in Germany (among which 2/3 had a Turkish migration background) and contradicted findings of a protective effect of spirituality and religious affiliation on bullying [65,66]. The analyses presented here showed that adolescents that engage in active bullying did so independently of their religiosity, i.e., they could not activate the aggression-regulative potential of religiosity, which is usually found in Muslim-majority countries [60,61]. Secondly, high religiosity was accompanied by a lower level of victimization and bullying–victimization [64], even taking into account several other factors, which seems to indicate that religiosity has a salutogenic effect [54,55,56] and acts as a buffer against highly negative experiences [58,59]. Thirdly, religiosity did not play any role in the bullying–depressiveness relationship. As we were not able to detect any buffering variables when testing ethnic class composition, acculturation strategies, and religiosity in a migration context, future studies could focus on the relationship between bullying experience and depressive symptoms in the context of resilience research [18,19,77]. Regarding religiosity as a potentially protective factor, we conclude that it does provide a buffer against the negative experience of victimization and bullying–victimization in the sample of adolescents from a Turkish migration background in Germany.

Despite the exploratory character of our study and the as yet non-existent validation of the measurements for this specific group of adolescents (due to the early stage of this research), we want to open a discussion about the salutogenic potential of religiosity and an integration acculturation strategy in the context of bullying prevention programs for adolescents with a (Turkish) migration background, which are considered a particularly vulnerable group [6,24]. This is especially important as policies, as well as intervention and prevention programs, against bullying usually neglect the specific protective factors in the context of migration. Such programs frequently address the problem of bullying by suggesting cooperation with educational personnel such as teachers [19,20], and community institutions such as after-school programs for private tutoring [19]. From the external side, cooperation with organizations, such as Turkish congregations and faith-based organizations like mosques, could also be included in this multilevel approach as religiosity seems to provide a buffer against victimization and bullying–victimization. The potential impact of this strategy seems to be significant, as the latter is usually associated with the highest rates of depressive symptoms and other psychological impairments [12]. Religiosity is an important component in the development of these adolescents [50,51,53] and is central to the formation of a stable system of norms and values, a main developmental task for adolescents [32].

In addition to the policy of encouraging children and adolescents with different ethnic minority backgrounds to develop a sense of ethnic identity and intercultural competency [3,24], new impulses for internal competencies that help cope with bullying experience can be made based on our results. Teachers can encourage and support their pupils of different ethnic backgrounds to develop integration as an acculturation strategy rather than using an assimilation approach, which is publicly evaluated as the best form of acculturation by mainstream European society [78]. Additionally, in the context of developmental psychology, initiating the development of integration as an acculturation strategy supports coping with identity formation as one of the main developmental tasks [31] among adolescents with a migration background. This should be implemented on the basis of a strong connectedness among the personnel at school and between the personnel and the pupils [19]. At the same time, this should also prevent marginalization tendencies, which are highly connected to depressive symptoms [40].

In order to strengthen the power of these impulses, it is essential that future studies, ideally with larger and more balanced samples, undertake further research on these two factors—integration and religiosity—in the context of bullying and migration. Such studies could then also clarify the transferability of the results and implications from adolescents with a Turkish migration background in Germany to other cultural contexts.

## 5. Conclusions

The present study showed empirical evidence for potentially protective factors against bullying experiences in a highly vulnerable group of adolescents: Youths with a Turkish migration background in Germany. While ethnic class composition did not show any effect, integration as an acculturation strategy, as well as religiosity, turned out to be protective factors against bullying experience. Although the character of this study is exploratory, and its results need to be tested further in future studies, it does suggest these factors have implications for bullying intervention programs. These programs could address the peculiarities of adolescents with a Turkish migration background, taking their general and migration-specific developmental tasks into consideration, for example, how they might form a stable system of (religious) values and identity in the context of migration.

## Figures and Tables

**Table 1:** Correlations between the included variables with means and standard deviations in brackets of the respective variable in the diagonal.

	1	2	3	4	5	6	7	8	9	10	11	12	13	14
Sociodemographic variables														
1 Age	16.17 (1.36)	−0.18	0.05	−0.07	−0.01	0.07	0.03	0.06	0.13	−0.20 *	0.03	0.01	−0.02	0.00
2 Gender (1 = female)		0.57 (0.50)	−0.11	0.23 *	−0.26 **	0.26 *	−0.02	0.08	0.08	0.01	0.03	0.01	0.04	0.11
3 Self-reported SES (0–4)			20.73 (0.72)	−0.01	0.11	0.06	0.12	−0.12	0.13	0.13	0.07	0.07	0.01	−0.16
4 Education (2–3)				2.76 (0.43)	−0.26 **	0.06	−0.14	−0.08	−0.08	−0.02	−0.16	−0.07	0.15	−0.03
Bullying experience (0–3)														
5 Bullying					0.46 (0.58)	0.18	0.80 **	0.23 *	0.04	0.09	−0.20 *	−0.06	−0.16	−0.14
6 Victimization						0.33 (0.51)	0.73 **	0.35 **	0.14	0.17	−0.22 *	−0.15	0.09	−0.39 **
7 Bullying–victimization							0.39 (0.42)	0.37 **	0.11	0.16	−0.27 **	−0.13	−0.05	−0.33 **
8 Depressive symptoms (0–5)								1.32 (0.89)	−0.01	0.02	−0.34 **	−0.07	0.29 **	−0.20 *
9 Ethnic class composition (0–4)									1.37 (1.46)	−0.02	−0.14	0.17	0.08	−0.06
Acculturation strategies (1–7)														
10 Assimilation										4.02 (1.12)	−0.10	−0.45 **	0.11	−0.47 **
11 Integration											5.19 (1.01)	0.09	−0.19 *	0.22 *
12 Separation												4.42 (1.20)	0.29 **	0.45 **
13 Marginalization													2.91 (1.25)	0.03
14 Religiosity (1–5)														4.33 (0.77)

*Notes*. * *p* ≤ 0.05, ** *p* ≤ 0.01, others not significant. SES = socioeconomic status. Numbers in brackets behind the variable/scale name indicate the range of the answer scale.

**Table 2:** Multiple regression analysis on bullying, victimization and bullying–victimization.

Dependent variables:	Bullying	Victimization	Bullying–Victimization
Age	−0.05	0.12	0.04
Female	−0.18	0.36 ***	0.09
SES	0.09	0.05	0.09
Education level	−0.22 *	−0.06	−0.19
Ethnic class composition	0.00	0.02	0.01
Assimilation	0.06	0.02	0.06
Integration	−0.26 *	−0.16	−0.27 **
Separation	0.07	0.04	0.07
Marginalization	−0.19	0.06	−0.10
Religiosity	−0.05	−0.40 ***	−0.28 *
*R^2^*	0.21 *	0.30 ***	0.21 *

*Notes*. * *p* ≤ 0.05, ** *p* ≤ 0.01, *** *p* ≤ 0.001, others not significant. SES = socioeconomic status.

**Table 3:** Multiple hierarchical regression analysis on depressive symptoms.

	1	2	3	4	5
Age	0.09	0.06	0.07	0.07	0.07
Female	0.10	0.05	0.06	0.12	0.15
SES	−0.18	−0.23 *	−0.22 *	−0.17 *	−0.18 *
Education level	−0.10	−0.06	−0.07	−0.20 *	−0.18
Victimization		0.14	0.14	0.04	−0.02
Bullying–victimization†		0.29 *	0.29 *	0.32 *	0.33 *
Ethnic class composition			−0.06	−0.09	−0.10
Assimilation				−0.15	−0.18
Integration				−0.19 *	−0.18 ^††^
Separation				−0.19	−0.14
Marginalization				0.37 ***	0.38 ***
Religiosity					−0.14
*R^2^_corrected_*	0.06	0.22 ***	0.22 ***	0.38 ***	0.39 ***
*R^2^_change_*	0.06	0.16 ***	0.00	0.16 ***	0.01

*Notes*. * *p* ≤ 0.05, *** *p* ≤ 0.001, ^††^ *p* ≤ 0.06, † Bullying alone is excluded from the analysis due to inter-collinearity with bullying–victimization.

## Dedication

This study is dedicated to Prof. Dr. Franz Petermann, who intensively contributed to its development. Prof. Petermann passed away on August 1st, 2019.
